# Neurotoxicity presenting as psychotic symptoms associated with calcineurin inhibitors: a narrative review with therapeutic perspectives

**DOI:** 10.1192/j.eurpsy.2026.11066

**Published:** 2026-07-31

**Authors:** M. G. Cabeza, C. Lopez, C. Cañete, I. Salmeron, E. Acosta

**Affiliations:** Hospital Clinic de Barcelona, Barcelona, Spain

## Abstract

**Introduction:**

Calcineurin inhibitors (CNIs), including tacrolimus and cyclosporine, are widely used immunosuppressants in transplant and autoimmune patients. Neuropsychiatric adverse effects are well described, yet psychotic presentations remain underrecognized and diagnostically challenging. Such cases are frequently misattributed to primary psychiatric disorders, delaying treatment and increasing the risk of complications. A recent clinical case of tacrolimus-associated psychosis prompted a review of the literature to summarize clinical features and management strategies.

**Objectives:**

To summarize the available evidence on neurotoxicity manifesting as psychotic symptoms in patients receiving CNIs, highlighting clinical features, risk factors, diagnostic findings, and pharmacological and non-pharmacological management.

**Methods:**

A bibliographic search of PubMed, Embase, and Cochrane (1990–2025) was conducted using keywords including calcineurin inhibitor, tacrolimus, cyclosporine, neurotoxicity, and psychosis. Reports describing psychotic or predominantly psychotic presentations temporally related to CNI exposure were reviewed. Data on patient characteristics, CNI dosing, timing of symptom onset, neuroimaging, treatment interventions, and clinical outcomes were extracted and summarized narratively.

**Results:**

Presentations ranged from acute confusional states with hallucinations to isolated psychotic syndromes with delusional ideation. Symptoms typically emerged days to months after CNI initiation. Elevated serum concentrations were common but not universal; some cases occurred at therapeutic levels, suggesting idiosyncratic or multifactorial toxicity. Neuroimaging occasionally revealed posterior reversible encephalopathy syndrome (PRES) or nonspecific white matter changes. Management generally involved dose reduction, discontinuation, or switching to non-CNI immunosuppressants, combined with short-term atypical antipsychotics (olanzapine, quetiapine, risperidone) and benzodiazepines. Most patients achieved resolution of psychotic symptoms following immunosuppressant adjustment.

**Conclusions:**

Although reversible, psychotic symptoms of CNI-related neurotoxicity are clinically significant. To strike a balance between neuropsychiatric safety and immunosuppressive efficacy, prompt detection and interdisciplinary management are crucial. Clinicians should emphasize the integration of psychiatric care into general medicine and remain vigilant for drug-induced psychosis in medically unwell patients.

**Disclosure of Interest:**

None Declared

